# O7-2 Structure and Trends in Preference of Individual Physical Activities in the Light of Physical Activity Recommendations

**DOI:** 10.1093/eurpub/ckac094.050

**Published:** 2022-08-29

**Authors:** Michal Kudlacek, Karel Fromel, Dorota Groffik

**Affiliations:** 1 Faculty of Physical Culture, Palacký University Olomouc, Olomouc, Czech Republic; 2 The Jerzy Kukuczka Academy of Physical Education, Akademia Wychovania Fizycznego, Katowice, Poland

**Keywords:** sport preferences, physical activity guidelines, IPAQ, monitoring

## Abstract

**Background:**

Regularly observed and sufficient physical activity (PA) of young people depends on the creation of conditions for success in the preferred PA. Therefore, we consider the diagnostics of PA preferences to be an irreplaceable part of PA diagnostics.

The aim of this study is thus to (a) detect the state and trends in the preferences of individually oriented PA of young people in different education and sports environments in the context of weekly PA; (b) to detect the associations among developing preferences of track and field and the fulfilment of recommendations within a weekly PA.

**Methods:**

In the research conducted from 2007 to 2017 participated in total 16116 participants aged from 14 to 26. We have realized a sports preferences questionnaire and weekly PA questionnaire IPAQ-long in order to detect the preferences in the individually oriented types of PA.

**Results:**

The biggest long-term stability among the Czech and Polish boys and the Czech girls showed swimming and cycling and among Polish girls swimming and skating. The most significant increase of preferences was detected in track and field, especially among the Czech girls and boys. The girls and boys who prefer track and field meet weekly PA recommendations significantly more than those who do not prefer it. Both Czech and Polish boys and girls showed that those who prefer athletic/running activities fulfil significantly more recommendations to a weekly PA; specifically at least 5 times a week for a minimum of 60 minutes of MVPA and simultaneously at least 3 times a week for a minimum of 20 minutes of vigorous PA. Preferences of athletic/running activities also increase the chance of fulfilment of above-mentioned recommendations to a weekly PA with both girls (OR = 1.801, CI = 1.571-2.065) and boys (OR = 1.655, CI = 1.437-1.905). These preferences are also important predictors for fulfilment of PA recommendations.

**Conclusions:**

The knowledge of trends in preferred types of PA has a predictive meaning for supporting physically active lifestyle of young people and for creation of optimal conditions to pursue popular types of PA.

